# Clinical effect analysis of timed strengthening moxibustion combined with continuing nursing on symptoms of qi deficiency and blood stasis type

**DOI:** 10.1097/MD.0000000000040886

**Published:** 2024-12-27

**Authors:** Fang Liu, Xuan Wang, Man Zhao, Qing Qin

**Affiliations:** a Rehabilitation Medicine, Huanggang Central Hospital, Huanggang, Hubei, China; b Department of Emergency Medicine, Huanggang Central Hospital, Huanggang, Hubei, China.

**Keywords:** clinical, nursing, qi deficiency and blood stasis, shoulder-hand syndrome, timed strengthening moxibustion, visual analogue scale

## Abstract

This study is based on the theory of meridian flow and aims to evaluate the clinical effect of timed strengthened moxibustion combined with continuous nursing on patients with shoulder-hand syndrome after stroke accompany with qi deficiency and blood stasis. This is a retrospective study. This study included 60 patients who visited the acupuncture and moxibustion Department of the local college of Chinese medicine from January 2022 to January 2023. They were separated into the experimental and controlling groups, 30 patients each. The clinical effect was evaluated through visual analogue scale, upper limb simplified Fugl-Meyer motor function score, Barthel index score, as well as other indicators. The treating period was 6 days per week, lasting for 2 courses. After treatment, the visual analogue scale score of the experimental group significantly decreased from 7.21 to 2.58 (*t* = 10.020, *P* < .01). Its upper limb simplified Fugl-Meyer motor function score significantly improved (*P* < .01). Its Barthel index score increased from 4.67 to 10.815 (*P* < .01), improving the daily living abilities. Its edema improved (Z = −2.898, *P* < .01), and its nursing satisfaction increased from 29.41% to 53.06% (*P* < .05). The combination of timed strengthening moxibustion and continuous nursing has a significant effect on treating the shoulder-hand syndrome in patients with qi deficiency and blood stasis type stroke. It can effectively reduce pain, improve motor function and daily living ability, and improve patient satisfaction with nursing, providing an effective treatment plan for post stroke rehabilitation.

## 1. Introduction

Shoulder-hand syndrome (SHS) is a common and challenging complication following stroke, affecting approximately 30% to 70% of stroke patients.^[1]^ This condition causes significant pain and functional impairment in the shoulder and hand, reducing patients’ quality of life and hindering rehabilitation efforts.^[2]^ SHS can also lead to secondary complications such as emotional distress, limited social participation, and increased dependence on care, which further complicates the recovery process.^[3,4]^

The underlying causes of SHS are multifactorial, involving brain damage, muscle weakness, and joint and tissue issues resulting from the stroke.^[5]^ Conventional treatments for SHS include physical therapy, drug therapy, and surgical interventions; however, each of these methods has limitations. For example, drug therapy may lead to side effects or dependency, while physical therapy and surgery often require specialized equipment and personnel, limiting accessibility for some patients.^[6,7]^

Moxibustion therapy, a traditional Chinese medicine technique, has gained attention as a potential treatment for SHS due to its noninvasive nature and effectiveness in promoting blood circulation, relieving muscle tension, and reducing pain.^[8,9]^ Unlike standard moxibustion, Timed strengthening moxibustion (TSM) intensifies the warming effect of moxibustion and applies precise timing to optimize therapeutic outcomes. TSM aims to activate qi and blood circulation more effectively and respond to individual needs by tailoring the timing and duration of moxibustion.^[4,10–12]^

This study explores the combined application of TSM and continuing nursing care for SHS in poststroke patients with qi deficiency and blood stasis (QDBS), with the goal of improving pain management, functional recovery, and daily activity capacity. This approach presents a promising alternative to conventional methods, providing a more targeted and holistic treatment option for poststroke SHS.

## 2. Materials and methods

### 2.1. General data

This study was a retrospective study approved by the Ethics Committee of Wuhan Hospital of Traditional Chinese Medicine. This study aims to probe the clinical efficacy of TSM and extended care in treating the poststroke SHS phase II patients with QDBS. The study lasted from January 2022 to January 2023. The subjects were 60 patients from the acupuncture and moxibustion Department of the local College of Traditional Chinese Medicine. These patients were strictly included in this study according to the inclusion criteria and were assigned to the Controlling Group (CG) and experimental group (EG), each having 30 people. On the basis of receiving routine treatment and care, CG received routine moxibustion therapy, while EG received TSM therapy at regular times. The treatment cycle for both groups was to rest for 1 day every 6 days after treating, with 7 days as a course, for a total of 2 courses. The study used a general information questionnaire to ensure baseline consistency between the 2 groups. The changes before and after treatment were evaluated using methods such as visual analogue score, Simplified Fugl-Meyer Motor Function Score (SFMFS) for upper limbs, Barthel index score for daily living activities, edema score, etc. All data were analyzed using SPSS 28.0 software. All patient guardians have signed informed consent forms. Table 1 presents the inclusion and exclusion criteria for cases.

**Table 1:** Case inclusion, exclusion, and shedding criteria.

Serial number	Standard	Standard content
1	Inclusion Criteria	It meets Western medicine and Traditional Chinese medicine’s diagnostic criteria for stroke
2		Age 40 to 80 years, male and female
3		Meets stage I diagnostic criteria for SHS staging and diagnostic criteria for symptoms of QDBS type
4		The course of the disease was between 2 weeks and 3 months, and the patient had the first onset of stroke
5		Vital signs were stable and well-conscious
6	Discharge criteria	A person with a history of mental illness
7		An allergic person
8		Pregnancy or have any other systemic severe disease
9		Previous history of periarthritis of shoulder, shoulder and hand pain

### 2.2. Research methods

The paper aims at analyzing the clinical effect of systematic strengthening of moxibustion combined with continuous care in the treatment of patients with QDBS. The sample size estimating method was used. According to an expected effective response rate of approximately 0.85 for patients, bilateral 0.05 was the significance level (a). The test efficacy was 0.80. The 2 sample mean comparison method in equation (1) was used. Where δ is the desired effect size, σ is the total standard deviation, and Zα and Zβ correspond to the bilateral significance level α and the standard normal values of the test efficacy, respectively.

$$\begin{array}{l} N=\frac{{\left(Z_{a}+Z_{\beta }\right)}^{2}\sigma^{2}}{\delta^{2}}(Q_{1}^{-1}+Q_{2}^{-1})\approx 50 \end{array}$$ (1)

Considering the possibility of patient loss, the sample size was increased by 20% to ensure the validity and reliability of statistical analysis, that is, 10 samples were increased, and finally 60 samples were collected. Through this method, the study aims to provide more accurate and comprehensive data to evaluate the impact of TSM combined with continuing care on QDBS.

### 2.3. Nursing methods

This study determined the sample size through a pilot study method, selecting 20% of patients as the pilot study sample. 12 patients were separated into 2 group, each having 6 people. Based on standard treatment and care, CG received routine moxibustion therapy, while EG underwent TSM, which was performed at a specific time (5–7 o’clock). Each moxibustion therapy lasted for 60 minutes, once a day. After 6 consecutive days of treatment, the patient rested for 1 day, for a total of 2 treatment courses. The 2 groups had the same procedure, with only differences in treatment time. This is aimed at reducing the bias of treatment time on outcomes.

For nursing interventions, both groups received basic treatment tailored to their condition, including circulatory improvement, neuroprotection, blood pressure control, blood glucose reduction, and lipid management. In addition, preventive measures such as avoiding pressure ulcers and lung infections were implemented, and a reasonable dietary plan was developed. Patients and their families received health education and psychological care, were guided on correct patient handling methods, and were prohibited from receiving intravenous fluids for the affected limb. And rehabilitation training nursing was implemented, including 45 minutes of active and passive exercise training once a day. The specific operations included shoulder joint, elbow forearm, wrist training, as well as BOBATH handshake exercises to promote the patient’s functional recovery.

The study was divided into CG group and EG group, and the 2 groups used different moxibustion methods on the basis of basic treatment and care. In this study, the control group (CG) received regular moxibustion treatment, while the EG received timed intensive moxibustion (TSM) treatment at a specific time window (5–7 o’clock). The treatment conditions, process and operation of moxibustion in the control group were the same as those in the EG, only the treatment time window was not limited. Specific operations are as follows:

1) Moxibustion process: The patient received moxibustion in a healthy lateral position, and selected the same acupuncture points (such as shoulder Anyu, arm 臑, Quchi, Hand Sanli, Hegu, Qi Hai, Blood Hai, etc) for moxibustion. Use a moxa stick with a diameter of 1.5 cm and a length of 20 cm, and fix it on the moxibustion rack after lighting, 2 to 3 cm away from the skin to avoid burns. Moxibustion was applied at each point for 60 minutes until the skin was slightly red and warm or slightly itchy.
2) Treatment environment: All patients are treated in an environment with moderate temperature and humidity to ensure patient comfort and comparability of efficacy. After treatment, the patient was observed for 15 minutes and then left.
3) Treatment cycle: Both the control group and the EG received continuous treatment for 6 days, with 1 day rest per week, for a total of 2 courses.

The treatment method for EG is TSM (5–7 o’clock). The moxibustion method is the same as CG, but all operations are performed from 5 to 7 o’clock.

Precautions and handling: Before implementing treatment, the research team will provide patients with a detailed introduction to the entire treatment process and operational details, ensuring that patients fully understand the treatment and coordinate the time with them. And patients are informed that if any discomfort occurs during treatment, they should immediately notify the doctor or nurse and have the right to terminate treatment at any time. Considering the long duration of moxibustion treatment, special attention should be paid to observing the skin reactions at the patient’s acupoints. If symptoms such as dizziness or palpitations occur during treatment, it should immediately stop treatment and take appropriate emergency measures, such as lying flat, raising the lower limbs, and giving warm sugar water. If the symptoms are not relieved, immediate first aid treatment is required. In addition, for possible skin problems such as blisters or minor burns, appropriate treatment will be taken, such as puncturing blisters, disinfecting, and applying burn ointment. If the burn is severe, it is necessary to seek medical treatment immediately.

### 2.4. Observation indicators

The study uses 4 evaluation indicators. These indicators aim to objectively and meticulously analyze the health transformation of patients from different dimensions.

(1) Pain assessment: Visual analogue scale (VAS) is used, ranging from 0 (painless) to 10 (severe pain), to assess patients’ limb pain. Its simplicity and ease to use provide convenience for its widespread clinical application.
(2) Upper limb motor function: SFMFS is used to assess the affected upper limb’s motor function. This scale has a total of 66 points, covering various aspects such as reflex activity, muscle coordination, and motor coordination, with higher scores indicating better the upper limb motor function.
(3) Daily living activity ability: Daily living activity ability is evaluated using the Barthel index, including daily living skills: eating, bathing, dressing, personal hygiene, and walking. The total score is 100 points, with higher scores indicating better daily living ability.
(4) Edema situation: According to the Chinese Rehabilitation Medicine Diagnosis and Treatment Standards, the degree of edema is divided into 4 levels, from no swelling (0 points) to severe swelling (3 points).

After treating 2 weeks, according to the efficacy evaluating criteria of the Rehabilitation Assessment and Treatment of Stroke, the efficacy is divided into 3 categories: significant, effective, and ineffective combined with specific patient conditions. The efficacy evaluation focuses on the improvement of joint pain and swelling, the degree of limited mobility, and muscle atrophy.

### 2.5. Statistical methods

This project uses SPSS 28.0 statistical software for relevant statistical analysis. The specific analysis methods are: (1) Gender count data: Chi square test is used to analyze the differences between gender count data. (2) Age, course of disease, VAS, SFMFS, and Barthel index score of daily living ability all conform to normal distribution, therefore *t* test is used for analysis. The results are presented in ($\bar{X}\pm S$). Independent and paired sample *t* tests are utilized for intergroup and intragroup comparing, respectively. (3) For data that does not follow a normal distribution, such as edema scores, nonparametric tests are used for analysis. (4) For overall efficacy level data, rank sum test is used for analysis. In statistical methods, when *P* < .05, the difference is considered statistically significant.

## 3. Results

### 3.1. Basic information

This study focused on patients with SHS stage I after QDBS stroke, each group having 30 cases To ensure the comparative effectiveness of the study, a detailed comparison was made between the basic information of 2 groups, including age, gender, and disease course.

Independent sample *t* test was used for comparing age. In Table 2, the 2 groups of patients showed good comparability in terms of age (*P* > .05). The chi square test method was utilized for comparing gender. The 2 groups are comparable at the gender level (*P* > .05). Regarding the comparison of disease course, the independent sample *t* test method was used. These 2 groups were comparable for disease course (*P* > .05).

**Table 2:** Basic information of patients.

Variable	CG (n = 30)	EG (n = 30)	*T/x* ^2^	*P*
Age	57.33 ± 6.81	59.33 ± 5.60	−1.239	*P* = .220 > .05
Gender			0.290	*P* = .589 > .05
Male	18	20		
Female	12	10		
Disease duration	55.53 ± 11.00	52.90 ± 11.51	0.906	*P* = .372 > .05

EG = experimental group.

### 3.2. Comparison of VAS scores

The comparison of VAS between these 2 groups before treating showed *P* > .05, demonstrating comparability in Table 3. In the intragroup comparison, compared to before treating, in CG, *t* = 11.049 and *P* < .01 after treating. In EG, *t* = 10.020 and *P* < .01. In inter group comparisons, the treatment plan showed statistically significant differences in reducing VAS. After treating 1 week (*t* = 2.998, *P* < .05) and 2 weeks (*t* = 2.998, *P* < .05), the effect of EG was superior to CG (*P* < .05).

**Table 3:** Visual analogue scale scores comparing.

Group	Pretreatment	1 week after	2 weeks after
CG (N = 30) (95%CI)	6.17 ± 1.09 (5.78–6.56)	4.83 ± 0.87 (4.52–5.14)	3.03 ± 1.00 (2.67–3.39)
EG (N = 30) (95%CI)	5.97 ± 1.22 (5.53–6.41)	4.47 ± 0.86 (4.16–4.78)	2.27 ± 0.98 (1.92–2.62)
*t*	0.672	2.998	2.998
*P*	.604	.004	.004

EG = experimental group.

### 3.3. Comparison of upper limb SFMFS

**Figure 1.**
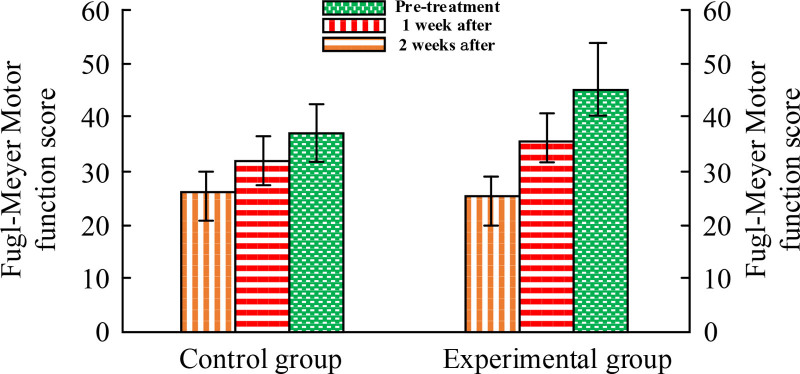
Comparison of upper limb simplified Fugl-Meyer motor function scores.

The SFMFS of the upper limbs before treatment was comparable between these 2 groups (*P* > .05) in Figure 1. In the intragroup comparison, the comparison between 1 week of treatment and before treatment showed *P* < .01. The comparing between 2 weeks and 1 week after treating also showed *P* < .01. In addition, in inter group comparison, the comparing of SFMFS in the upper limbs between these 2 groups showed *P* < .01. EG showed better therapeutic effects compared to CG. This indicates that the combination of TSM and continuing care has a significant therapeutic effect (*P* < .01).

### 3.4. Comparison of Barthel index scores

**Figure 2.**
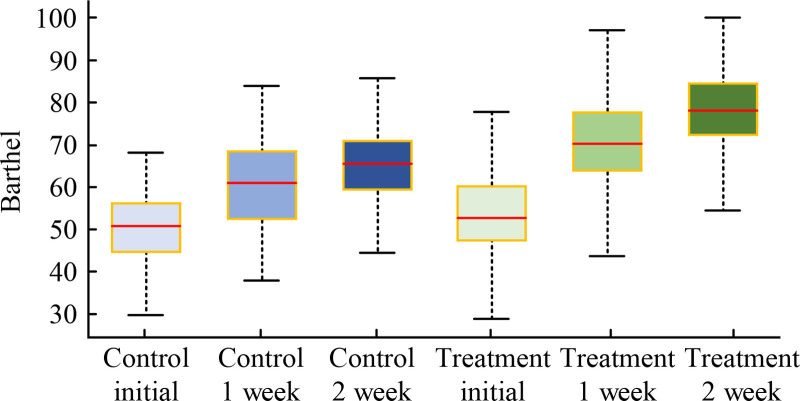
Comparison of Barthel index scores.

In Figure 2, these 2 groups’ comparison of Barthel index scores before treatment showed *P* > .05, proving comparability. After treating 1 week, the Barthel index of both groups improved, with significant improvements in CG (*t* = −7.210, *P* < .01) and EG (*t* = −14.317, *P* < .01). Continuing to compare the changes between these 2 groups after 1 week of treatment and before treatment, CG had significant changes (*t* = −8.603, *P* < .01) and EG (*t* = −13.358, *P* < .01). After treating 2 weeks, the Barthel index scores of CG (*t* = −8.267, *P* < .01) and EG (*t* = −10.815, *P* < .01) were compared, showing a sustained and statistically significant therapeutic effect (*P* < .01).

### 3.5. Comparison of edema scores

The edema situation of the 2 groups before treatment showed *P* > .05 through nonparametric testing, ensuring comparability of patients in Table 4. The comparison of EG between 1 and 2 weeks and before treatment showed *P* < .05. When comparing 2 weeks to 2 week, the comparison between CG and EG showed *P* < .01. In the intergroup comparison, EG showed statistical differences in reducing edema compared to CG (*Z* = −2.202, *P* < .05 after 1 week, and *Z* = −2.898, *P* < .01 after 2 weeks), demonstrating the advantage of EG in reducing QDBS symptoms.

**Table 4:** Comparison of edema scores.

Group	Pretreatment	1 week after	2 weeks after
CG (N = 30)	2 (2,3)	2 (1,3)	1 (1,2)
EG (N = 30)	2 (2,3)	2 (1,2)	1 (0,1)
*Z*	−0.449	−2.202	−2.898
*P*	.664	.030	.004

EG = experimental group.

### 3.6. Comparison of overall efficacy

**Figure 3.**
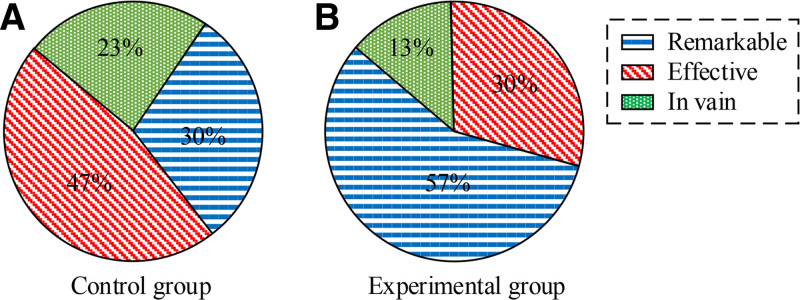
Comparison of overall efficacy.

Figure 3 presents a rank sum test 2 groups’ overall efficacy after treatment. After treating 2 weeks, 2 groups’ clinical efficacy comparison showed *P* < .05. This emphasizes the effectiveness of TSM combined with continuous care in treating QDBS symptoms.

### 3.7. Comparison of nursing satisfaction

**Figure 4.**
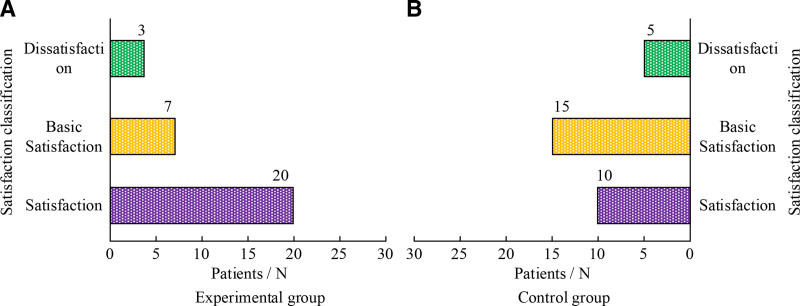
Comparison of nursing satisfaction.

In terms of patient satisfaction, the overall satisfaction of EG patients with nursing care was significantly better than CG’s (*P* < .05), highlighting the effectiveness of TSM and its continued care in improving patient satisfaction with nursing care. Figure 4 shows the relevant detailed data.

## 4. Discussion

In the clinical treatment of poststroke SHS, an important direction of current medical research includes exploring effective pain management and functional recovery strategies. SHS is a common complication of stroke, characterized by shoulder pain and hand dysfunction, which seriously affects the rehabilitation process and patients’ life quality.^[13,14]^ The combination of traditional medicine and modern treatment methods has gradually become an important treatment strategy for improving symptoms of SHS. Among numerous treatment methods, TSM combined with continuous care is an innovative approach that combines traditional moxibustion with modern care, adjusting treatment frequency and time to achieve better treatment outcomes.^[15,16]^ Traditional moxibustion therapy typically involves a moxibustion duration of 15 to 30 minutes and is considered a standard practice for providing mild and effective therapeutic effects. However, for certain chronic diseases and complex clinical situations, the duration of traditional moxibustion therapy may not be sufficient to achieve the desired therapeutic effect, such as QDBS type poststroke SHS. Therefore, the heavy moxibustion method has emerged, which combines traditional moxibustion methods with increased moxibustion volume to provide more in-depth and lasting therapeutic effects. Its moxibustion time usually exceeds 30 minutes and can reach 60 minutes or longer, thereby enhancing the intensity and duration of thermal stimulation, which more effectively promotes local blood circulation and metabolic processes.^[17–19]^ TSM further developed on the basis of heavy moxibustion, providing personalized treatment for the patient’s qi and blood circulation patterns by precisely controlling the time and frequency of moxibustion application. This method not only prolongs the duration of moxibustion treatment, but also precisely adjusts the timing of moxibustion application to better meet the patient’s biological rhythm and treatment needs. TSM aims to achieve comprehensive and in-depth treatment of patient symptoms through continuous thermal stimulation and precise time control. This method can not only effectively alleviate pain, but also promote the functional recovery of damaged limbs, especially showing positive effects in promoting patients’ daily activity. In addition, the implementation of continuing care provides patients with continuous medical support and health guidance, helping them maintain treatment effectiveness in the later stages of treatment and prevent symptom recurrence. This continuous care model emphasizes the overall needs of patients, strengthens their self-management ability by providing personalized health education and rehabilitation training, and provides a solid foundation for their long-term rehabilitation. In summary, the combination of TSM and continuing care has significant implications for the treatment of SHS patients after QDBS stroke. This method not only provides targeted pain relief and functional recovery, but also plays an important role in improving patients’ self-care ability and quality of life.

In the evaluation of clinical efficacy, significant improvement in pain perception of EG after treatment can be observed through comparative analysis of VAS. The average visual simulation score of EG decreased from baseline 7.21 to 3.45 (*P* < .001) after 1 week of treatment, and further decreased to 2.58 (*P* < .001) after 2 weeks. Compared to CG, the pain relief effect of EG1 week was more significant (VAS decreased to 4.67 after CG1 week, *P* < .5), indicating that TSM is superior to CG in reducing VAS and improving pain. Pain usually originates from poor qi and blood flow, and moxibustion effectively reduces pain through its warming effect and promoting blood flow. This treatment method combines the concept of meridian flow injection, enhances the therapeutic effect, achieves the goal of promoting blood circulation, removing stasis, and relieving pain. For the recovery of upper limb motor function, SFMFS provides a quantitative tool for research to measure treatment effectiveness. Patients with EG showed significantly better recovery in this score compared to CG (*P* < .01). This indicates that TSM plays an effective role in pain control and a crucial role in improving patient motor function. In terms of improving daily living activities, this therapy can activate specific acupoints in a balanced manner through the warm and deep stimulating effects of moxibustion, promote the recovery of body metabolism and neurological function, and help restore daily living abilities. It can achieve optimal results when treated at scheduled times. The improvement of Barthel index score reveals the positive effect of TSM on improving patients’ daily living abilities. After 1 week of treatment, both groups showed an improvement in Barthel index, especially in EG, which showed a more significant improvement (*P* < .01). Regarding the edema score, this method involves generating heat and light effects, penetrating deep into the skin and even the body, promoting blood circulation and lymphatic flow in affected areas, reducing inflammatory reactions, and effectively reducing swelling. The edema improvement of EG was significantly better than that of CG after 1 week and 2 weeks of treatment, showing statistical differences of *Z* = −2.202 (*P* < .05) and *Z* = −2.898 (*P* < .01), respectively. This once again confirms the effectiveness of TSM in promoting blood circulation and reducing edema. The comparison of overall efficacy further emphasizes the advantages of TSM. The overall efficacy of CG was 9, 14, and 7, respectively, with a total effective rate of 76.67%, while that of EG was 17, 9, and 4, with a total effective rate of 86.67%. The rank sum test results (*P* < .05) after treating 2 weeks indicate statistical significance of the treatment. In addition, the survey results of nursing satisfaction reflect the satisfaction and recognition of patients towards nursing services, which is crucial for hospitals to improve nursing services and enhance patient rehabilitation outcomes. In this study, the nursing satisfaction rate of EG patients was significantly higher at 53.06% than that of CG29.41% (*P* < .05), highlighting the effectiveness of TSM and its continued care in improving patient nursing satisfaction. Based on these results, the combination of TSM and continuing care has shown significant advantages in pain relief, motor function recovery, daily living ability improvement, edema reduction, and overall efficacy improvement for patients with poststroke SHS of QDBS type. And by improving patient satisfaction with nursing care, this treatment method has also contributed to the overall recovery and improvement of the patient’s life quality.

In summary, the combination of TSM and continuing care has shown significant clinical effects in the treatment of SHS after QDBS type stroke. It not only has clear benefits in symptom relief and functional recovery, but also has significant advantages in patient care satisfaction. Future research can further explore the potential application of this method in other types of traditional Chinese medicine symptoms. And it can strengthen the theoretical research and clinical application of timing treatment methods for midnight-noon ebb-flow, providing more comprehensive and personalized treatment plans for stroke patients.

## Author contributions

**Conceptualization:** Fang Liu, Xuan Wang, Man Zhao, Qing Qin.

**Data curation:** Fang Liu, Xuan Wang, Man Zhao, Qing Qin.

**Formal analysis:** Xuan Wang, Qing Qin.

**Funding acquisition:** Qing Qin.

**Investigation:** Fang Liu, Xuan Wang.

**Methodology:** Fang Liu, Xuan Wang, Man Zhao.

**Supervision:** Xuan Wang, Man Zhao, Qing Qin.

**Validation:** Fang Liu, Man Zhao, Qing Qin.

**Visualization:** Fang Liu.

**Writing – original draft:** Fang Liu, Xuan Wang, Qing Qin.

**Writing – review & editing:** Fang Liu, Xuan Wang, Qing Qin.
